# Supplementary material for: Brassica napus Roots Use Different Strategies to Respond to Warm Temperatures
Source: Int J Mol Sci. 2023 Jan 6;24(2):1143. doi: 10.3390/ijms24021143 (PMC9863162; doi:10.3390/ijms24021143)
Supplement: Supplementary file 1 [file ijms-24-01143-s001.zip › Figure S1 IJMS_2022.pdf]

A

Extent traits

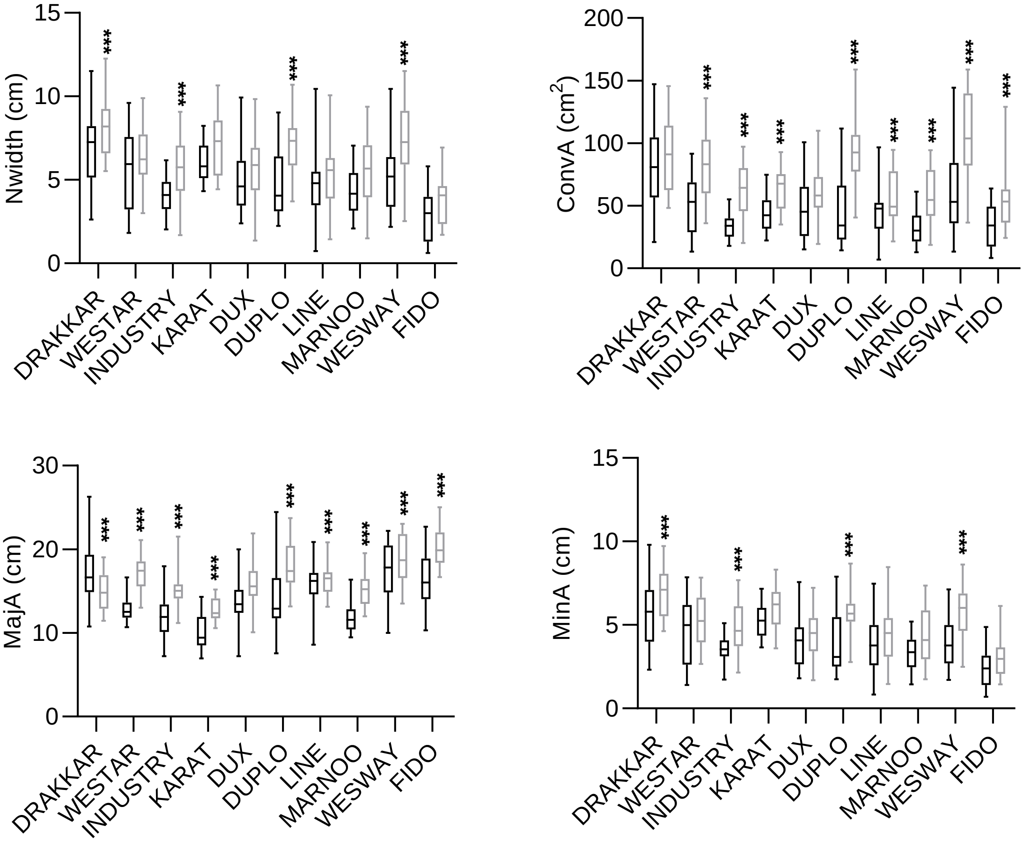

B

Size traits

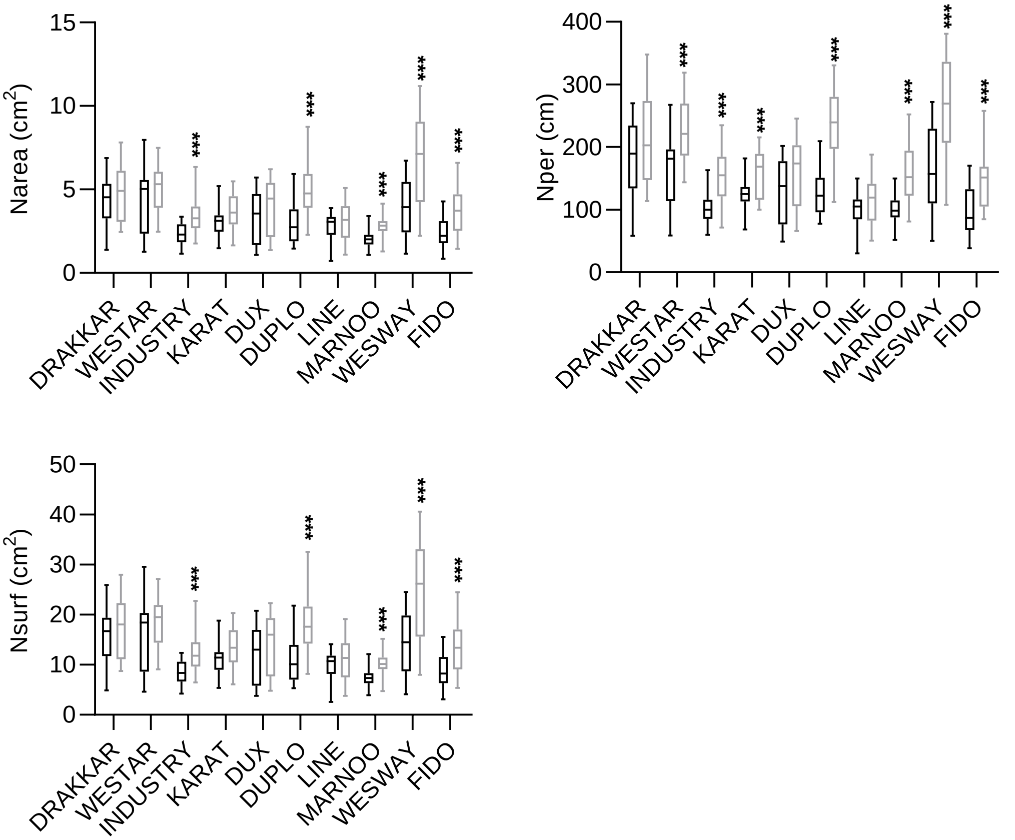

C

Distribution traits

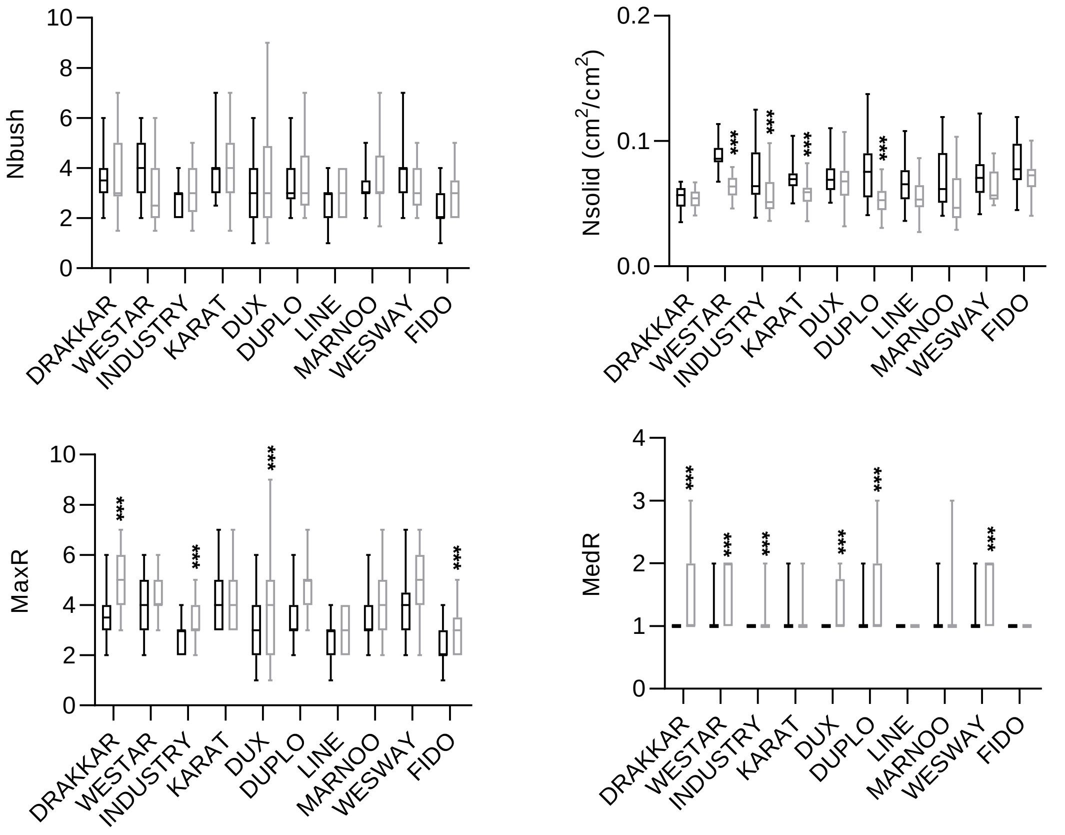

D

Secondary roots traits

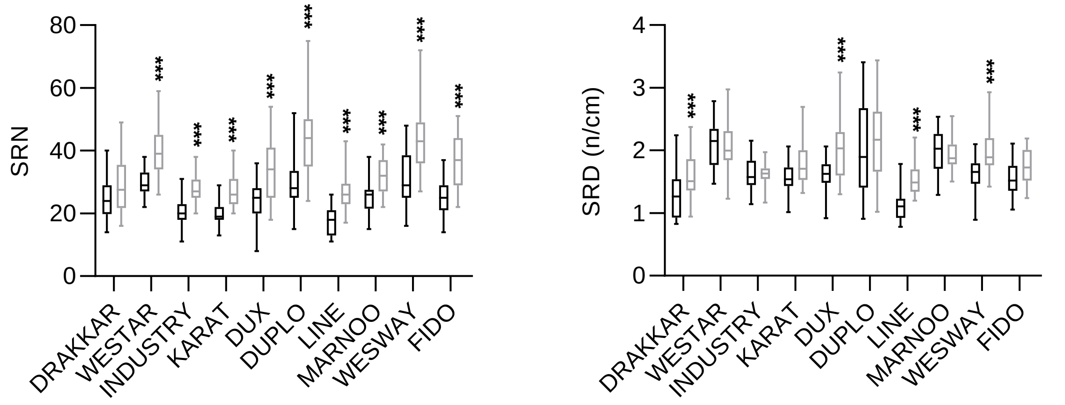

E

Shape traits

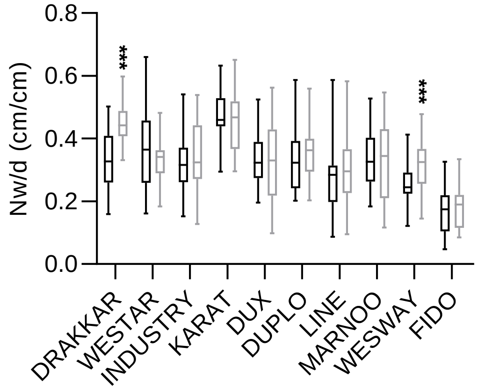

Figure S1. Warm conditions provoke extensive changes in all major root traits in *B. napus*. Com-parative values of root extent-related traits **(A)**, size-related traits **(B)**, distribution-related traits **(C)**, secondary roots traits **(D)** and shape-related traits **(E)** of a collection of 10 SOSR genotypes grown at 21ºC or 29ºC. Statistical t-test analysis, *** FDR<0.01.
