# Supplementary material for: Brassica napus Roots Use Different Strategies to Respond to Warm Temperatures
Source: Int J Mol Sci. 2023 Jan 6;24(2):1143. doi: 10.3390/ijms24021143 (PMC9863162; doi:10.3390/ijms24021143)
Supplement: Supplementary file 1 [file ijms-24-01143-s001.zip › Figure S2 IJMS_2022.pdf]

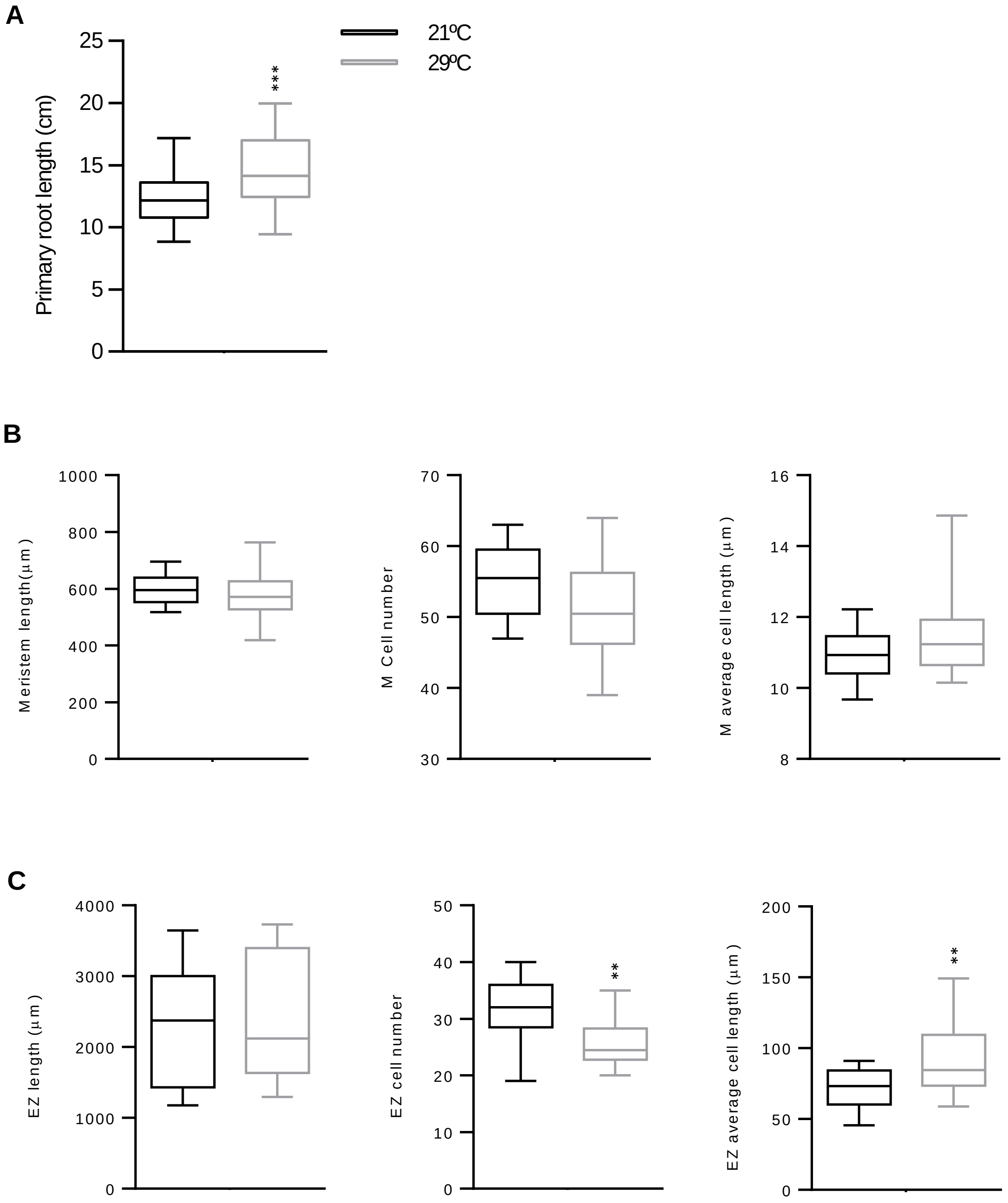

Figure S2. Cellular response of Marnoo primary root to warm temperatures. **(A)** Primary root length of Marnoo seedlings grown 7 days at 21ºC and 29ºC. Statistical t-test analysis, ***$p<0.0001$. **(B)** Meristem length, cell number and average cell length of cells in the meristematic zone of Mar-noo grown at 21ºC and 29ºC. Statistical t-test analysis found no significant differences. **(C)** Elongation zone length, cell number and average cell length of cells in the elongation zone of Marnoo grown at 21ºC and 29ºC. Statistical t-test analysis, ** $p<0.001$.
