# Supplementary material for: Brassica napus Roots Use Different Strategies to Respond to Warm Temperatures
Source: Int J Mol Sci. 2023 Jan 6;24(2):1143. doi: 10.3390/ijms24021143 (PMC9863162; doi:10.3390/ijms24021143)
Supplement: Supplementary file 1 [file ijms-24-01143-s001.zip › Figure S3 IJMS_2022.pdf]

A

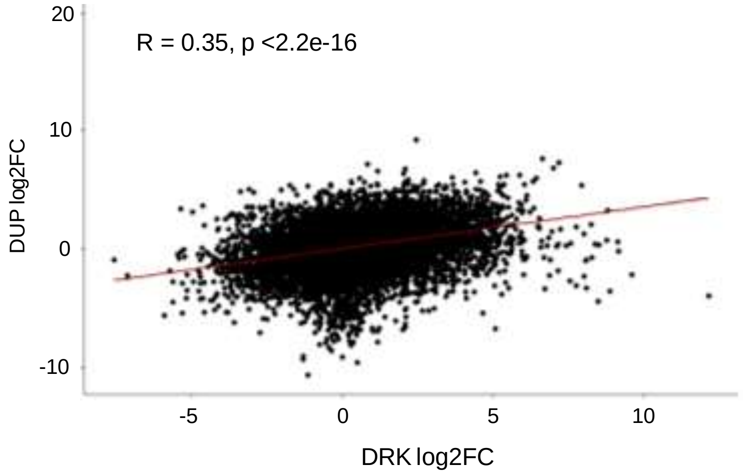
R = 0.35, p <2.2e-16
20
10
0
-10
DUP log2FC
-5
0
5
10
DRK log2FC

B

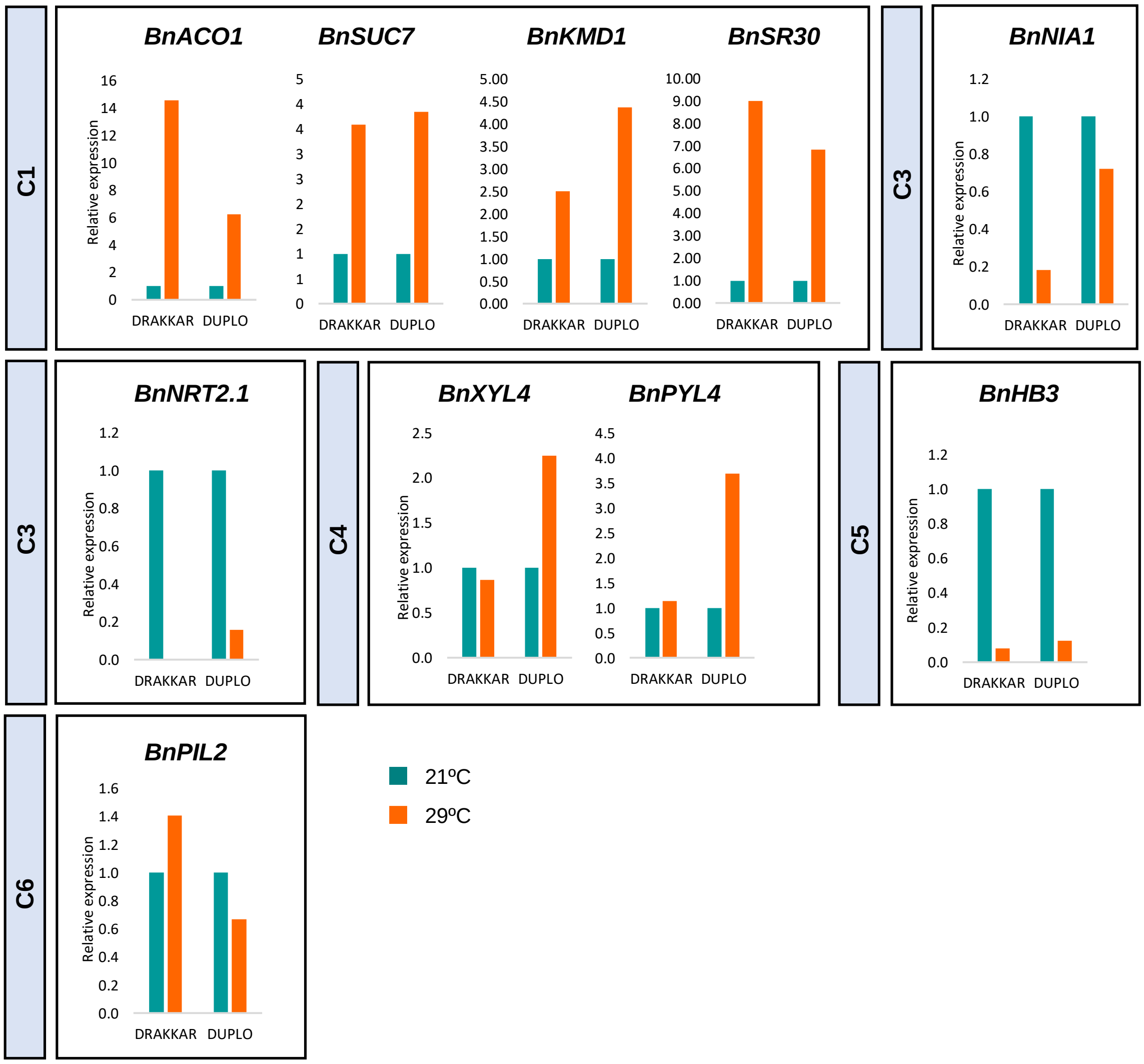
C1
BnACO1
BnSUC7
BnKMD1
BnSR30
C3
BnNIA1
C3
BnNRT2.1
C4
BnXYL4
BnPYL4
C5
BnHB3
C6
BnPIL2
Relative expression
DRAKKAR
DUPLO
21ºC
29ºC

c

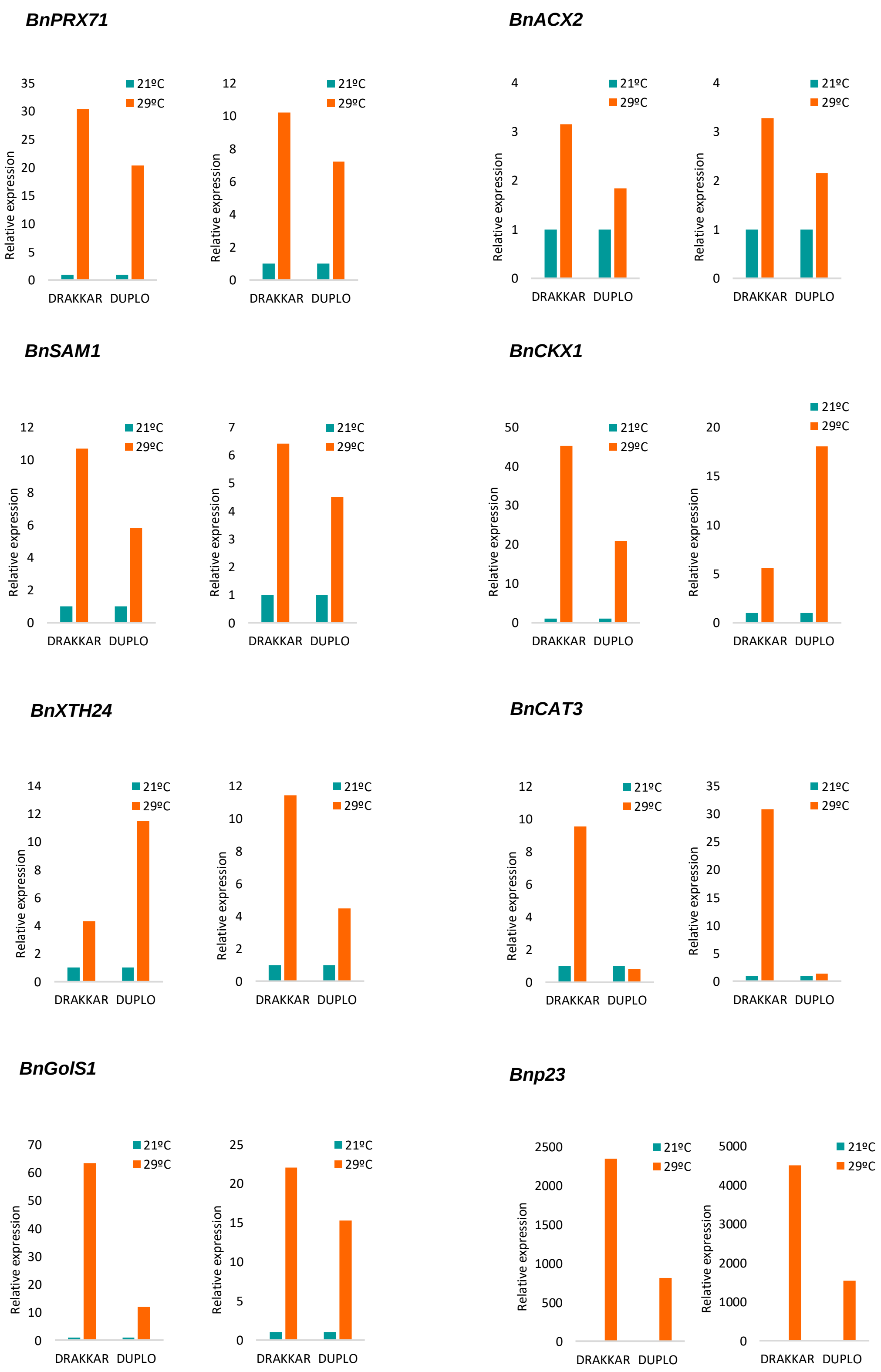
BnPRX71
BnACX2
BnSAM1
BnCKX1
BnXTH24
BnCAT3
BnGolS1
Bnp23
21ºC
29ºC
Relative expression
DRAKKAR
DUPLO
0
5
10
15
20
25
30
35
2
4
6
8
12
1
3
7
40
50
60
70
500
1000
1500
2000
2500
3000
4000
5000

BnHSA32

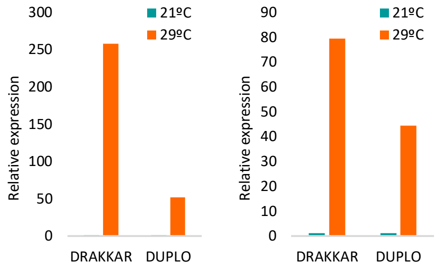

BnHSP15.7

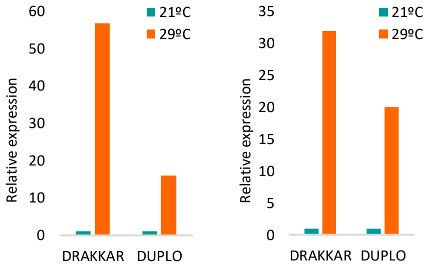

BnXTR6

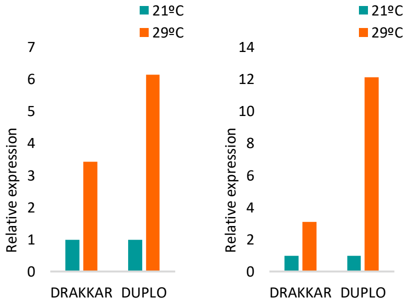

BnEXPA8

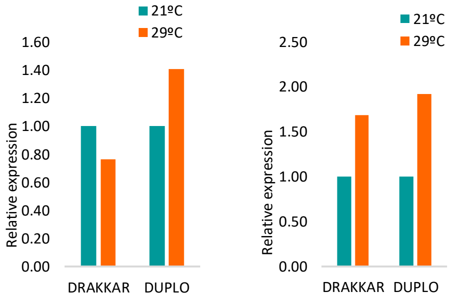

BnPYL5

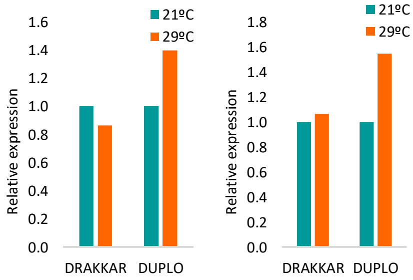

BnSTE1

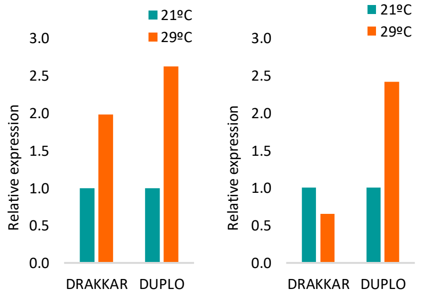

BnGA2OX2

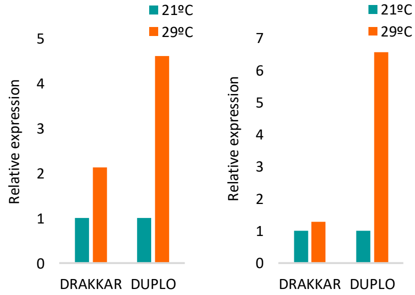

BnSR30

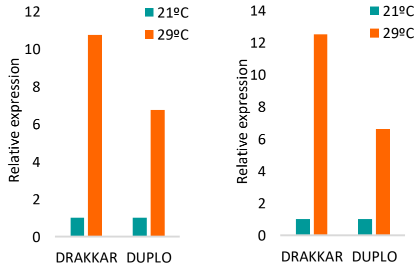

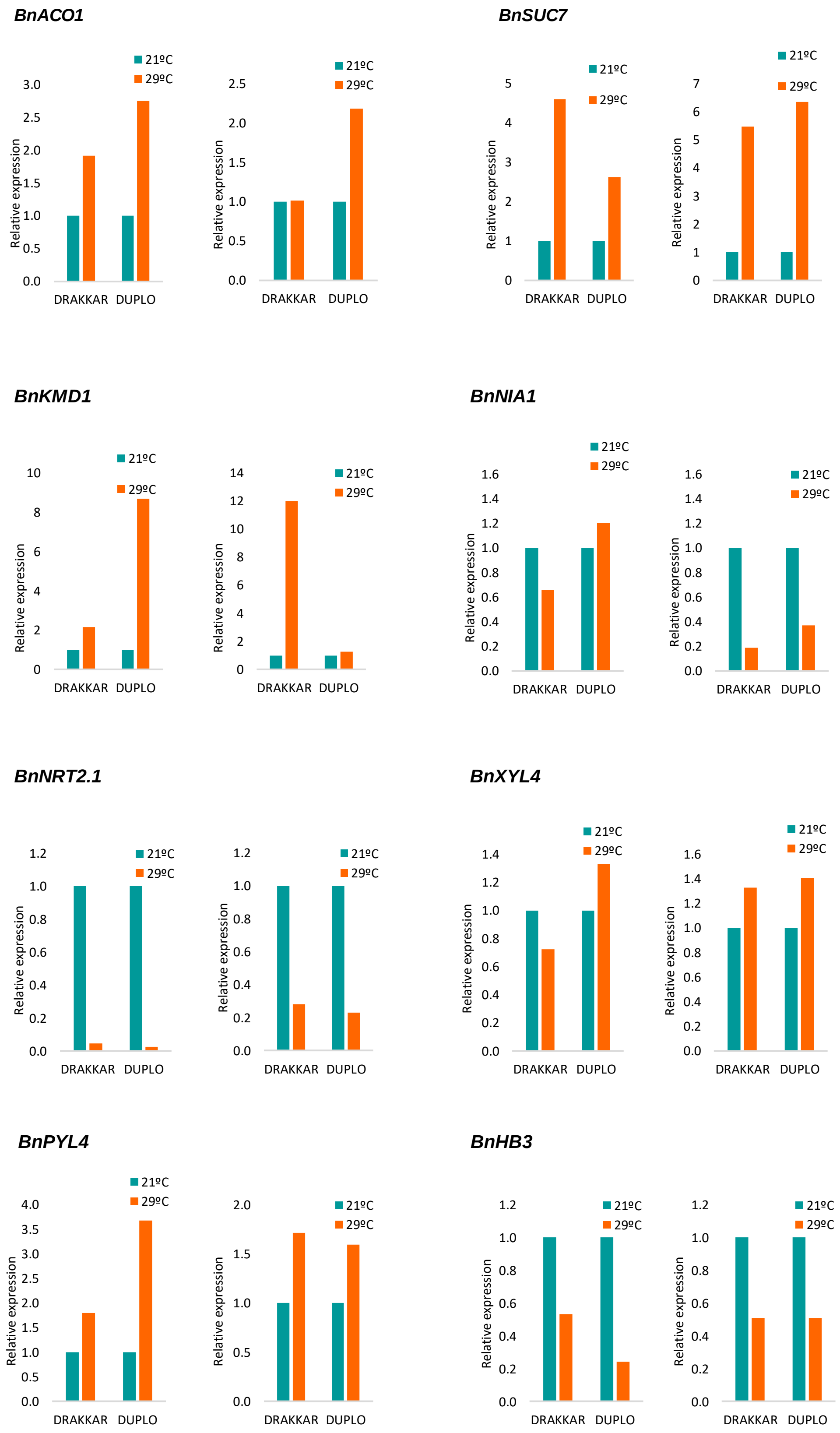
BnACO1
BnSUC7
BnKMD1
BnNIA1
BnNRT2.1
BnXYL4
BnPYL4
BnHB3
21ºC
29ºC
Relative expression
DRAKKAR
DUPLO

BnPIL2

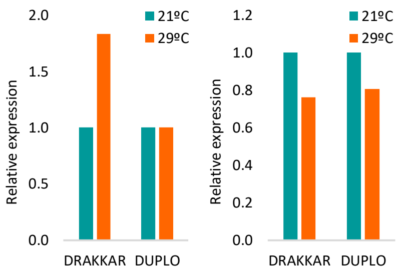

Figure S3. Differential gene expression patterns determine the variability of root response to warm temperature in B. napus. **(A)** Correlation analysis of gene expression between Drakkar and Duplo root tips grown at 21ºC and 29ºC. **(B)** Relative gene expression values of genes, as meas-ured using quantitative RT-PCR (qPCR), from Cluster 1: related with ethylene (ET) biosynthesis genes, BnACO1; mechanisms of temperature sensing and signalling like sugar transport, BnSUC7, and splicing machinery, BnSR30 and degradation of CKs, BnKMD1; from Cluster 3: related with nitrate assimilation and transport, BnNIA1 and BnNRT2.1; from Cluster 4: related with cell wall biogenesis and organization, BnXYL4 and ABA sensing, BnPYL4; from Cluster 5: related to CK signalling, BnHB3; and from Cluster 6: related to negative regulation of photomorphogenesis, BnPIL2, identified by hierarchical clustering analysis of Drakkar and Duplo root tips grown at 21ºC compared to 29ºC. The second and third biological replicates can be found in Figure. S2C. **(C)** Second and third biological replicates of qPCRs of DEGs between Drakkar and Duplo root tips grown at 21ºC and to 29ºC from all the clusters analysed. The first biological replicates are shown in Figure. 6 and Figure. S3B.
